# Predicting antibiotic resistance in Enterobacterales to support optimal empiric treatment of urinary tract infections in outpatient veterans

**DOI:** 10.1017/ash.2024.377

**Published:** 2024-09-09

**Authors:** Ben J. Brintz, Karl Madaras-Kelly, McKenna Nevers, Kelly L. Echevarria, Matthew B. Goetz, Matthew H. Samore

**Affiliations:** 1 Division of Epidemiology, Department of Internal Medicine, University of Utah, Salt Lake City, UT, USA; 2 IDEAS Center, VA Salt Lake City Healthcare System, Salt Lake City, UT, USA; 3 College of Pharmacy, Idaho State University, Meridian, ID, USA; 4 Pharmacy Service, Boise VA Medical Center, Boise, ID, USA; 5 Pharmacy Benefits Management Program, U.S. Department of Veterans Affairs, Hines, IL, USA; 6 David Geffen School of Medicine at UCLA, Los Angeles, CA, USA; 7 VA Greater Los Angeles Healthcare System, Los Angeles, California, USA

## Abstract

**Objective:**

Bacterial resistance is known to diminish the effectiveness of antibiotics for treatment of urinary tract infections. Review of recent healthcare and antibiotic exposures, as well as prior culture results is recommended to aid in selection of empirical treatment. However, the optimal approach for assessing these data is unclear. We utilized data from the Veterans Health Administration to evaluate relationships between culture and treatment history and the subsequent probability of antibiotic-resistant bacteria identified in urine cultures to further guide clinicians in understanding these risk factors.

**Methods:**

Using the XGBoost algorithm, a retrospective cohort of outpatients with urine culture results and antibiotic prescriptions from 2017 to 2022 was used to develop models for predicting antibiotic resistance for three classes of antibiotics: cephalosporins, fluoroquinolones, and trimethoprim/sulfamethoxazole (TMP/SMX) obtained from urine cultures. Model performance was assessed using Area Under the Receiver Operating Characteristic curve (AUC) and Precision-Recall AUC (PRAUC)

**Results:**

There were 392,647 prior urine cultures identified in 214,656 patients. A history of bacterial resistance to the specific treatment was the most important predictor of subsequent resistance for positive cultures, followed by a history of specific antibiotic exposure. The models performed better than previously established risk factors alone, especially for fluoroquinolone resistance, with an AUC of .84 and PRAUC of .70. Notably, the models’ performance improved markedly (AUC = .90, PRAUC = .87) when applied to cultures from patients with a known history of resistance to any of the antibiotic classes.

**Conclusion:**

These predictive models demonstrate potential in guiding antibiotic prescription and improving infection management.

## Introduction

The efficacy of antibiotic treatment for urinary tract infection (UTI) is in part dependent on the susceptibility of the infecting pathogen; however, prescribing decisions are often made empirically before culture results become available. There is a need for tools that can support a clinician’s ability to select effective empirical therapy. As UTIs usually result from colonization with exogenous or endogenous Gram-negative flora; prior hospitalization, antibiotic exposure, and previous culture of antibiotic-resistant organisms are known risk factors for subsequent culture of multi-drugresistant organisms (MDRO).^1–8^ In contrast, clonal spread of resistance determinants may result in pathogens that are resistant to antibiotics that the patient has not previously received.^9,10^ Several tertiary references recommend that prior hospitalization, antibiotic exposure, or prior antibiotic resistance to commonly utilized UTI treatments within the past three months is an indication for initial therapy with a carbapenem or aminoglycoside.^6–8^ The optimal approach to utilizing these risk factor data is unclear. The Veterans Health Administration (VHA) is the largest integrated healthcare system in the United States with over 9 million Veterans, for which antibacterial prescription and susceptibility data are captured electronically.^11^ Therefore, VHA data offers unique insight into the study of the temporal relationship between antibiotic exposure and bacterial resistance.

To provide further insight into the relationships between these risk factors and subsequent antibiotic resistance we evaluated susceptibility profiles of Enterobacterales from urine cultures collected from veterans in the outpatient setting. These data were used to 1) examine the relationship between past antibiotic class exposures, prior antibiotic resistance identified through culture, and subsequent urine culture susceptibility profiles to enhance understanding of antibiotic selective pressure, and 2) develop predictive models to support choice of empiric antibiotics pending results of susceptibility testing. In particular, we sought to predict the probability that a pathogen recovered from urine culture was susceptible (or resistant) to any of three commonly utilized classes of antibiotics utilized to treat UTIs: fluoroquinolones (FQ), trimethoprim/sulfamethoxazole (TMP/SMX), or cephalosporins (cephs).

## Methods

**Figure 1.**
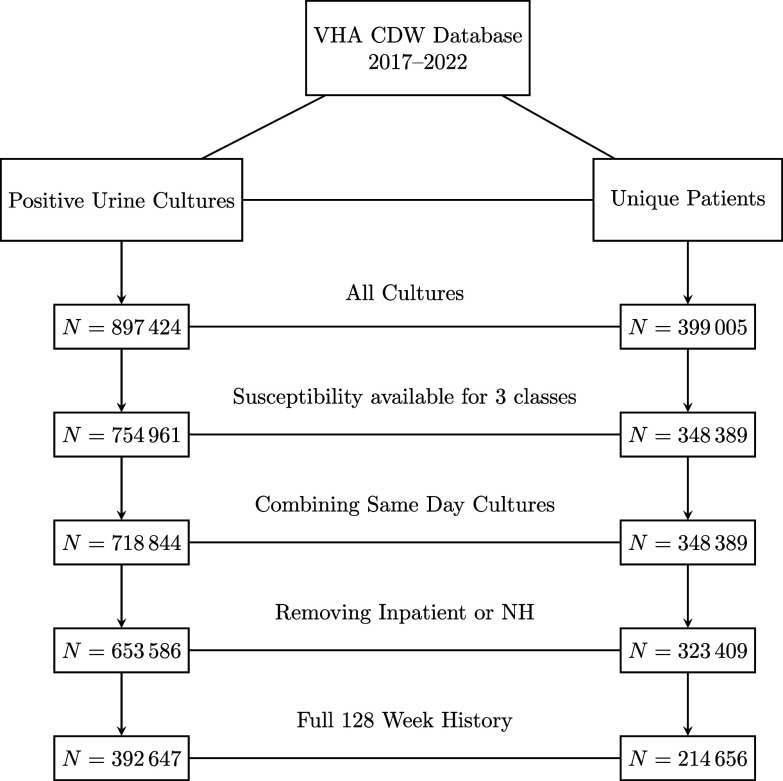
Consort diagram showing number of urine cultures and number of unique patients with urine cultures at each step of data cleaning. CDW, Corporate Data Warehouse; NH, Nursing Home.

A retrospective cohort of veterans with urine culture results obtained between January 2015 and October 2022 with outpatient antibiotic prescription data of interest available was developed. For each culture, we identified organisms of interest including *Escherichia coli* (*E. coli*), Citrobacter spp., Enterobacter spp., Klebsiella spp., Proteus spp., and Serratia spp., and established whether they were susceptible (S), intermediate (I), or resistant(R) to ceph (any reported generation), FQ, and TMP/SMX. To predict resistance on subsequent culture in the outpatient setting, we evaluated cultures for all patients (inpatient and outpatient settings) with susceptibility results available for the three antibiotic classes of interest. For each qualifying culture, we identified a collection date. If two positive cultures were collected from a patient on the same day they were combined and counted as resistant if at least one of the cultures was resistant (Figure 1). For each antibiotic, values of susceptible or intermediate were classified as susceptible to create a binary variable.

The following data elements were extracted from the VHA Corporate Data Warehouse: patient demographics (age, sex, and Charlson comorbidity index), culture collection date, organism identification and semi-quantitative sensitivity results, outpatient antibiotic prescription history, dates and types of healthcare exposures including acute care admissions and nursing home stays.^11,12^ We connected each subsequent (i.e., index) culture to patient demographics, treatment history, and patient-specific positive and negative urine culture history.

Treatment history was defined as the number of prescriptions the patient received for each antibiotic class within weekly intervals prior to the positive index urine culture. Similarly, a negative culture history variable was based on the number of negative cultures, i.e., cultures that came back with no organisms identified collected in identical time intervals. Finally, the positive culture history variable included a count of resistant positive cultures in one feature and count of susceptible positive cultures in another feature for ceph, FQ, and TMP/SMX prior to the index positive urine culture. The weekly intervals used to classify variables have been shown to capture the effect of patients’ history on the subsequent development of resistance.^13^

The following covariates were considered to develop each model: prior inpatient stay within the last 90 or 365 days, prior nursing home stay within the last 90 or 365 days; age, sex, Charlson Comorbidity Index, date by quarter for trend, quarter alone for seasonality, history of antibiotic prescription (set A), history of negative cultures (set A), and positive culture history (set B) (Table 1).

**Table 1.** Week intervals used for summarizing antibiotic prescription and culture collection history

**Set A:** Antibiotic prescription and negative culture history (Weeks)	0–1, 2–4, 4–8, 8–16, 16–32, 32–64, 64–128
**Set B:** Positive culture history (Weeks)	1–2, 2–4, 4–8, 8–16, 16–24, 24–32, 32–40, … 112

We used the XGBoost algorithm, an ensemble approach that uses gradient boosted decision-trees to derive predictive models for estimating the probability that a pathogen recovered from culture was resistant to each ceph, FQ, and TMP/SMX antibiotic class.^14^ The importance of each predictor using built-in XGBoost output which calculates the fractional contribution of each predictor to the model based on the total gain in performance of the predictors’ decision tree splits was utilized. A higher percentage indicates a more important predictor. Finally, Shapley Additive Explanations (SHAP), a value that expresses the additive contribution of each feature to the predicted value, was used to evaluate the direction and magnitude of main effects and to assess the interactions between having a history of resistant or susceptible urine culture and having had an antibiotic treatment.^15^ Interactions were assessed by calculating the average change in the SHAP values based on the presence of treatment in each interval dependent on the presence or absence of a resistant or susceptible culture in each interval (Table S1).

The generalizable performance of each model was estimated by training on a random 80% of data and tested on the remaining 20%. Before fitting a model on full training data, five-fold cross-validation within the training set to tune hyperparameters: max depth, *η* (learning rate), and number of rounds (trees). Area under the receiver operating characteristic curve (AUROC or AUC) was used to assess performance for both cross-validation, model fitting, and importance metrics. To assess performance on the test set, precision-recall (PR) curves, representing the trade-off between sensitivity and positive predictive value (PPV), and the area under the PR curves were calculated. Using precision, or PPV instead of specificity is particularly useful in the presence of an imbalanced outcome when a strong specificity is more easily achievable due to the smaller rate of positive outcomes. ROC curves and PR curves were compared to the sensitivity and specificity as well as sensitivity and PPV which could be achieved on test set patients based on previously published MDRO risk factors^6–8^ for selection of empirical antibiotic treatment for UTI including:

1. Antibiotic-resistant Gram-negative urinary isolate cultured in the prior 3 months
2. Recent (3 months) specific antibiotic exposures (i.e., FQ, TMP/SMX, ceph)
3. Recent (3 months) inpatient stay (i.e., hospital, nursing home, and long-term acute care facility)

Each model was assessed for calibration, first by estimating the calibration intercept and slope as well as by fitting smoothed curves to show the relationship between estimated risk and observed proportion of events.^16^ All analyses were conducted using R version 4.1.0^17^ using the XGBoost package.^18^ This research complies with all federal guidelines and Department of Veterans Affairs policies relative to human subject research.

## Results

The cohort included 392,647 positive urine cultures obtained from 214,656 unique patients with a complete treatment history obtained between 2017 and 2022. Patients were elderly, predominantly male, and had moderate co-morbidity (Table 2). The majority (54%) of positive urine cultures grew *Escherichia coli* (Table S2). Of those cultures, 14.9, 27.4, and 24.5 percent were resistant to ceph, FQ, and TMP/SMX, respectively.

**Table 2.** Summary of unique patients and prior exposures

Characteristic	*N* = 214,656
Age(years), Mean (S.D.)	68.9 (14.8)
Male sex [*N*, (%)]	167,439 (78.0)
Charlson Comorbidity Index, Median (IQR)	2.00 (0, 4)
**Positive Urine Cultures Per Patient**	
Mean (S.D.)	1.83 (1.70)
Median (IQR)	1.00 (2.00, 4.00)
**Prior Antibiotic Resistance [** ***N*** **, (%)]**	
None	243,053 (61.9)
Cephalosporin	32, 981 (8.4)
Fluoroquinolone	26,879 (6.8)
TMP/SMX	28655 (7.3%)
Cephalosporin & Fluoroquinolone	10,808 (2.8)
Cephalosporin & TMP/SMX	7,592 (1.9)
Fluoroquinolone & TMP/SMX	22,951 (5.8)
Cephalosporin, Fluoroquinolone, TMP/SMX	19,728 (5.0)
**Exposure to Antibiotic Class in prior 128 weeks, [N, (%)]**	
Cephalosporin	113,262 (28.9)
Fluoroquinolone	109,020 (27.8)
TMP/SMX	97,707 (24.9)
**Healthcare Exposure, [N, (%)]**	
Prior Inpatient Stay in last 90 days	53,399 (13.6)
Prior Nursing Home Stay in last 90 days	4,004 (1.0)
Prior Inpatient Stay in last 365 days	111,904 (28.5)
Prior Nursing Home Stay in last 365 days	10,954 (2.8)

**Figure 2.**
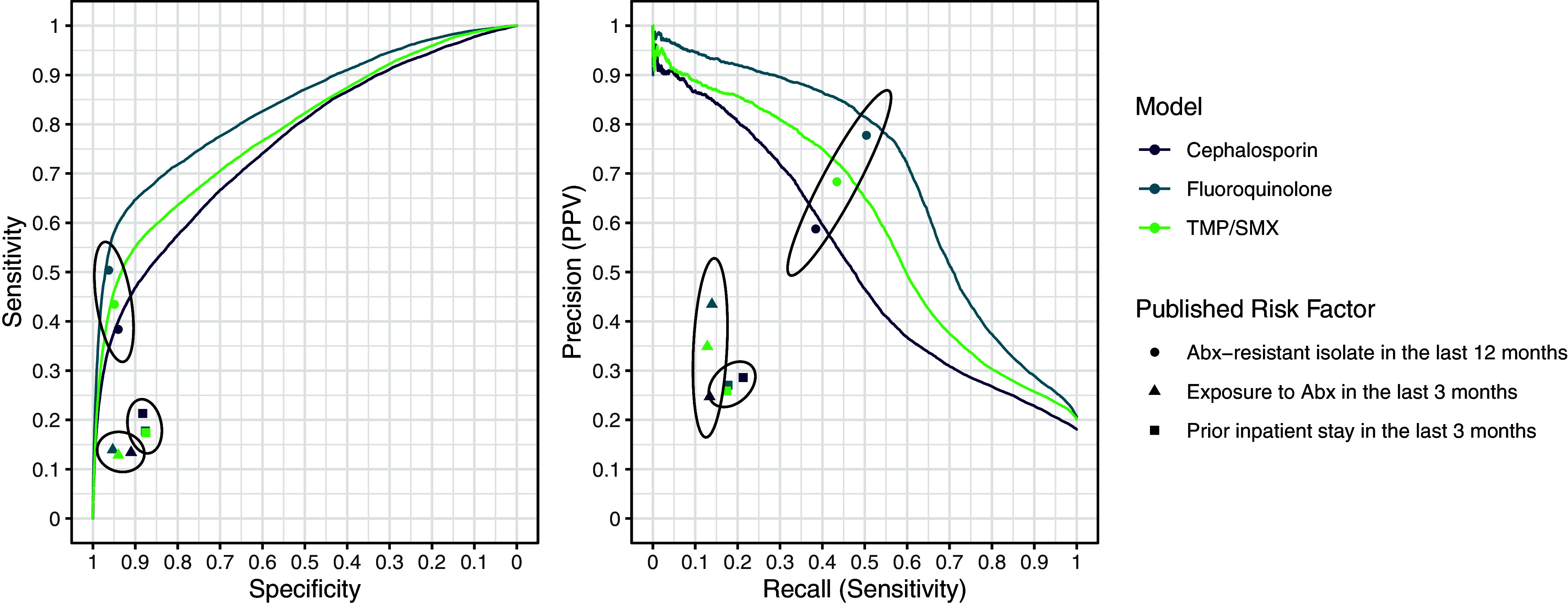
Receiver operating characteristic (Left) and precision-recall (Right) curves for each predictive model. Trade-off between sensitivity and specificity (Left) and precision and recall (right). Individual points represent performance of referenced risk criteria for exposure or isolation specific to each resistance outcome by color.

Based on tuning from cross-validation on the training set, an XGboost model with 30 trees, a max depth of 7, and a learning rate (*η*) of .3 was fit for predicting the probability that an index culture was resistant for each of the antibiotic classes of interest. When applied to test data, model AUCs were .76, .84, and .79 and PRAUCs were .52, .70, and .60 for ceph, FQ, and TMP/SMX, respectively (Figure 2). At a negative predictive value of .90, the ceph model achieved a PPV of .35, a specificity of .75, and sensitivity of .63, the FQ model achieved a PPV of .72, a specificity of .94, and a sensitivity of .60, and the TMP/SMX model achieved a PPV of .41, a specificity of .76, and a sensitivity of .67. At a lower negative predictive value of .82, the PPV for all three antibiotics improved (ceph .93, FQ .93, TMP/SMX .88), the specificity is 1.00, but the sensitivity drops (ceph .01, FQ .15, TMP/SMX .13). The distribution of predictions on the test set for each model was right-skewed and uni-modal for antibiotic susceptible cultures but was bimodal for resistant cultures indicating strong specificity but not necessarily strong sensitivity or negative predictive values (Figure S1). The ROC and PR curves demonstrate that the XGBoost-derived models outperformed MDRO risk factor criteria in terms of sensitivity, specificity, and PPV (Figure 2). The same is true when the MDRO risk factor criteria were compared for any or all ceph, FQ, or TMP/SMX previous resistant isolates or antibiotic exposures for each resistance outcome (Figure S2).

All three models achieved weak (i.e. not over- or underestimating risk) calibration with calibration intercepts close to 0 and calibration slopes close to 1 (Figure S3).

A history of antibiotic resistance to the specific treatment was the most important predictor of subsequent resistance for positive index cultures, followed by a history of specific antibiotic exposures. The next most influential variable for FQ and TMP/SMX models was patient age with a 96% relative decrease in importance from the most important variable. The next most influential variable in the ceph model was the Charlson Comorbidity Index with a 90% relative decrease in importance.

**Figure 3.**
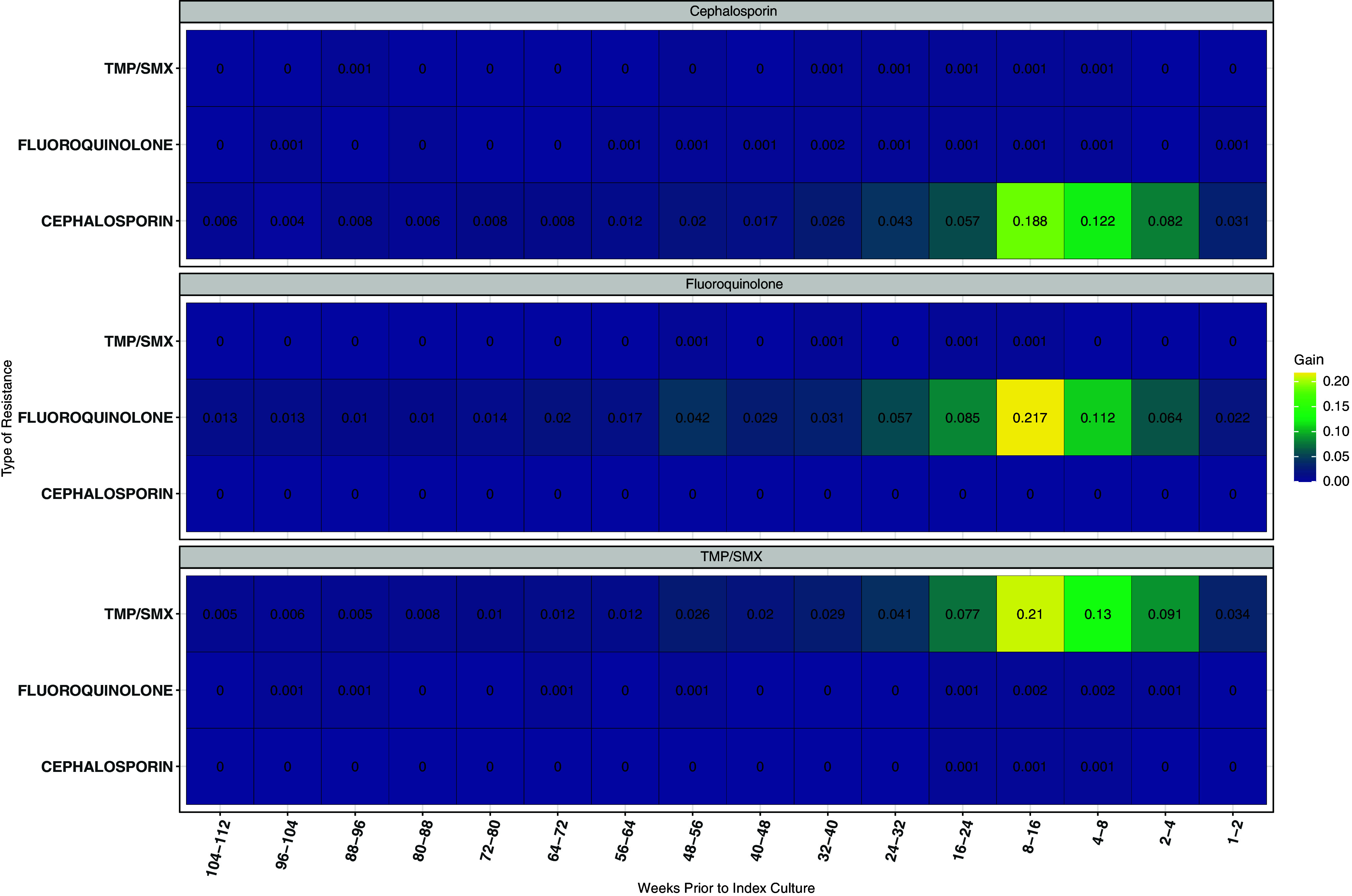
Optimal time-window in weeks for prior antibiotic resistance to predict subsequent culture of antibiotic resistance isolates. The week interval x-axis represents the presence of a resistant sample in weekly periods of study. The color and the annotated text represent the gain in area under the receiver operating characteristic curve when the variable is included in the predictive model.

**Figure 4.**
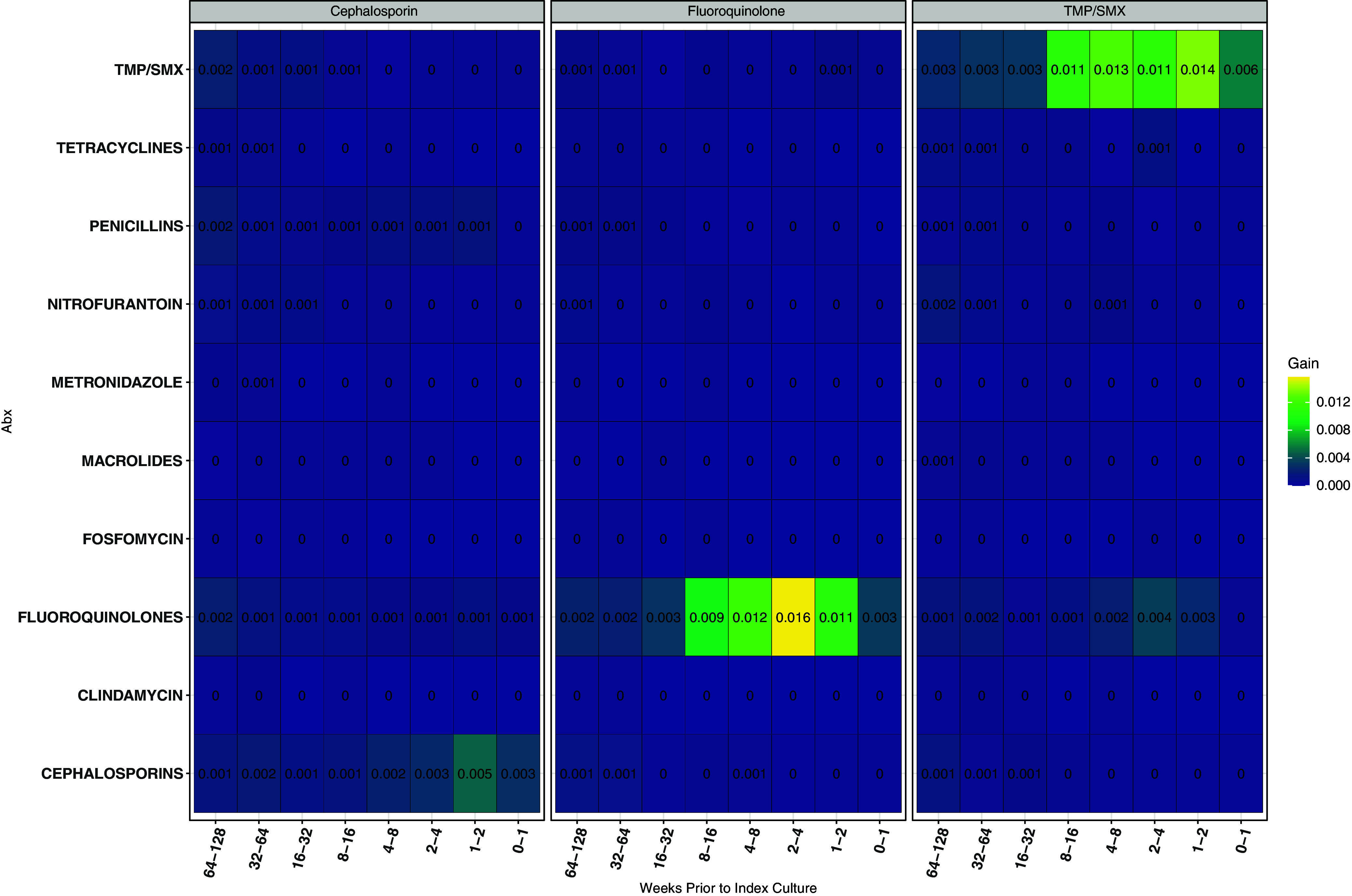
Heat plot presenting the importance of treatment history of a range of antibiotics for predicting the outcomes. The x-axis represents the presence of a resistant isolate in culture at weekly interval. The color and the annotated text represent the gain in AUC when the variable is included in the predictive model.

A history of antibiotic resistance between 8 and 16 weeks prior to index culture was the most important predictor of resistance for all three models (Figure 3). The interval of 1 to 8 weeks prior contained the most important predictors related to treatment history with the specific antibiotic class on index culture susceptibility (Figure 4). Neither Figure 3 nor Figure 4 suggest evidence of prior resistance or antibiotic prescription influencing subsequent antibiotic resistance to a different class on index culture.

**Figure 5.**
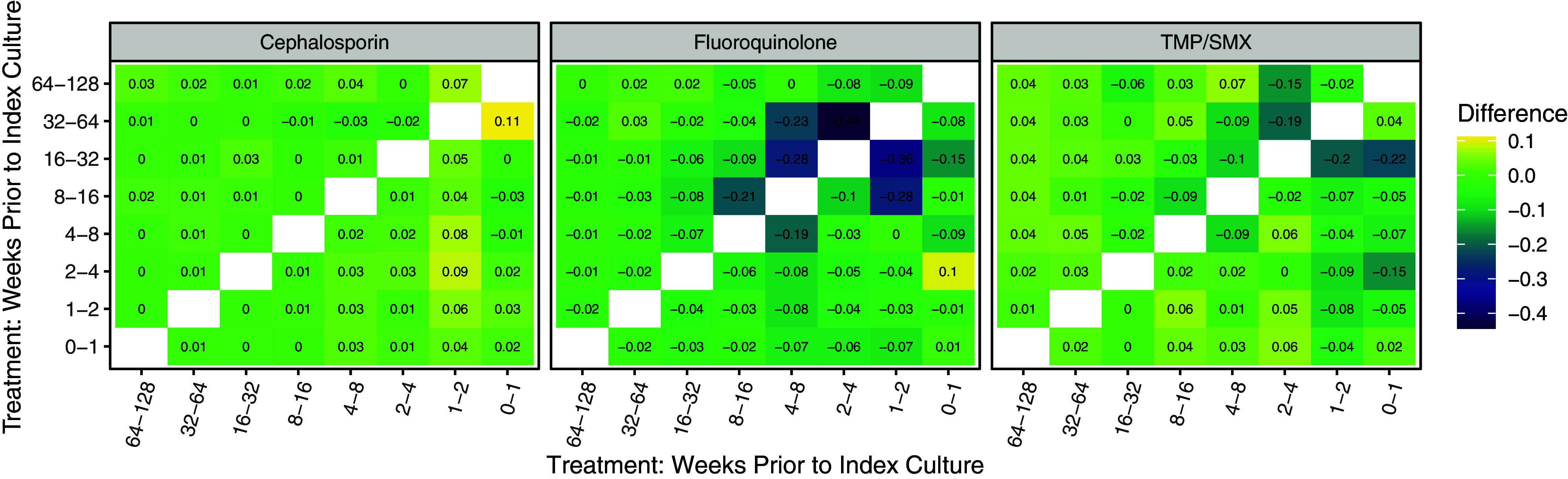
Heat plot presenting the difference in average SHAP values for antibiotic exposed versus unexposed cases by week interval (x-axis), according to whether or not there is a history of the same antibiotic class exposure in a different weekly interval (y-axis). The color represents the magnitude of difference, i.e., the interaction effect. Blue suggests that the effect of the treatment on the prediction is lessened among individuals who have a history of treatment during a different week interval. In some circumstances, predictive power was reduced when individuals received treatment during multiple time intervals. eg, the -.44 in the middle panel at the 4 on the x-axis and 2 on the y-axis suggests that the difference in average Shapley Additive Explanations value between those having received fluoroquinolones (FQ) treatment 2–4 weeks prior and not having received FQ treatment 2–4 weeks prior decreases by .44 in those who also had treatment with FQ 1–2 weeks ago.

The SHAP value represents the contribution of prior antibiotic exposure and resistance history to the predicted value for the full model (Figure S4). All plots indicate the variables have a positive effect on the prediction of subsequent resistance. The smallest contribution occurred with prior ceph exposure or ceph resistance, and the largest contribution occurred with prior FQ exposure or resistance which is consistent with Figures 4 & 5. Finally, there is evidence of an interaction between each prior treatment and history of a resistant or susceptible culture to the prescribed treatment (Figure S5). The contribution of treatment to the prediction is lessened when there is a history of a resistant culture and increases when there is history of a susceptible culture. The size of the interaction is less for ceph than it is for either FQ or TMP/SMX. The impact of prior cumulative antibiotic class exposures across multiple time intervals does not appear to be additive to the subsequent identification of resistance in the index culture (Figure 5). This decrease in effect dissipates as the treatment time intervals increase. As seen previously, this interaction effect is less strong in ceph.

In a post-hoc analysis, the predictive performance restricted to cultures with any history of susceptibilities reported (i.e., any isolate with susceptibilities reported for ceph, FQ, or TMP/SMX) achieved AUC of .83, .91, and .87 and PRAUC of .65, .83, and .73 for ceph, FQ, and TMP/SMX models, respectively. This provides strong evidence that the models perform better when used to predict resistance for positive urine cultures with any prior history of resistance (*P*-value *<* .0001).

## Discussion

Successful management of bacterial infections depends on prescribing antibiotics that are active against the causative pathogen. Models to predict the probability of resistance have the potential to improve the empiric choice of antibiotics. Our approach extends previously published models of resistance in urinary pathogens in several key ways.^13,19,20^ We created a longitudinal dataset from VA electronic health records that was national in scope, representing diverse populations and multiple types of outpatient settings. Novel predictors in our analysis included history of negative urine cultures and history of urine cultures that yielded bacteria resistant to drugs other than the target antibiotic. We used SHAP values to interpret the relative contribution of different variables to the predictive model and to explore interactions.

Our models confirm that recovery of antibiotic-resistant organisms in prior cultures, followed by antecedent antibiotic exposure, are the most important predictors for antibiotic resistance to ceph, FQ, and TMP/SMX. We found that the model of FQ resistance had the highest accuracy, followed by TMP/SMX, then ceph. Similarly, prior treatment with FQ was a better predictor of FQ resistance than TMP/SMX was for TMP/SMX resistance, which was stronger than for ceph treatment and ceph resistance. The time window for which treatment had its maximal predictive power was 1-4 weeks prior to the index culture. However, for all three outcomes, a history of prior antibiotic resistance during the time window of 8 -16 weeks was more predictive of resistance than history of resistance during the time window of 1-7 weeks. This surprising finding should be examined in other patient populations.

Another striking finding was that history of prior resistance to antibiotics other than the target class was far weaker as a predictor than history of resistance to the target antibiotic class, despite the correlation between susceptibility results for ceph, FQ, and TMP/SMX. Moreover, exposures to antibiotics other than the target antibiotic class were relatively not predictive of resistance to the target drug. Similar results have been previously reported.^13^ The lack of evidence of co-selection may at least in part be explained by the built-in regularization of the XGBoost algorithm which can diminish the importance of the final prediction of a feature in a collinear set.

Our models outperform the published risk factor criteria for UTI treatment, though it is important to recognize that the prior risk factors were originally developed to identify patients with likely MDRO and not necessarily select appropriate treatment.^6–8^ Similar to other studies, we found that prior exposure in healthcare settings, which is sometimes used as a proxy for history of resistance, had low predictive power.

SHAP scores are useful for quantifying the explanatory power of individual features and for exploring how a feature’s predictive power depends on the values of other features. However, they should not be interpreted as estimations of causal effects. Using SHAP values to explain the magnitude and direction of feature effects, there was evidence of interaction between a history of treatments in different intervals and a history of resistant or susceptible culture. Both Figure S4 and Figure 5 suggest that treatments do not have additive contributions within or between intervals, respectively. They also suggest that the combination of a history of susceptible form of an organism plus treatment with the target antibiotic is predictive of the emergence of resistance.

These models condition on the presence of a positive culture. Therefore, its potential usefulness for clinical decision-making would be to guide therapy during the interval from when it is known that a urine culture is positive to the time that susceptibility results are reported. Our models could be combined with a model that predicts whether the culture is positive to guide antibiotic choice at the time the urine specimen is collected, in the situation where empiric treatment is warranted. Due to the models’ calibration, they are best used as a risk prediction for which a clinician determines a course of action based on the continuous risk value (Figure S3). This would allow the clinician to choose their own risk threshold, which is often dependent on the patient’s morbidity.

The study has several limitations. First, as stated above, the predicted outcome is antibiotic resistance, given a positive urine culture. As a result, this prediction can not be used before the determination that the urine culture has yielded an enteric Gram-negative rod. However, it is feasible to develop a predictive model for a positive urine culture test and to condition based upon a positive urine culture prediction. Further research is needed to assess the effect of conditioning on a previous prediction. Second, our analysis of antibiotic exposure did not include treatments administered in acute care hospitals or nursing homes or cultures or treatments received outside of the VHA system, a more general limitation of integrating prediction models into an electronic medical record. Finally, we did not compare different machine learning algorithms. Some methods, such as the random forest conditional permutation algorithm, have been developed to be robust to collinearity when measuring importance. However, this algorithm is memory-intensive and computationally intractable for this data in the VHA system. In conclusion, we developed a decision-making tool that incorporates a patient’s history in order to guide antibiotic prescription. We demonstrate that these tools, especially the FQ model, have strong discriminatory performance and predict risk that reflects observed event rates. Such a predictive model can potentially improve the management of infection and guide the use of antibiotics in the VHA.

## Supporting information

Brintz et al. supplementary material Brintz et al. supplementary material

## Supplementary material

For supplementary material accompanying this paper visit http://doi.org/10.1017/ash.2024.377. click here to view supplementary material

## Research Transparency and Reproducibility

All data for our study were accessed by means of the Veterans Health Information Systems and Technology Architecture, through the Veterans Informatics and Computing Infrastructure, a computing environment that stores clinical data for research purposes.
