# Supplementary material for: Predicting antibiotic resistance in Enterobacterales to support optimal empiric treatment of urinary tract infections in outpatient veterans
Source: Antimicrob Steward Healthc Epidemiol. 2024 Sep 9;4(1):e118. doi: 10.1017/ash.2024.377 (PMC11384162; doi:10.1017/ash.2024.377)
Supplement: Brintz et al. supplementary material [file S2732494X24003772sup001.docx]

# Supplemental Tables and Figures

**Tables**

**Table S1: Summary of average SHAP values used to calculate interactions between features.**

| **Treatment** | **Resistant Culture** | **Average SHAP value** |
| --- | --- | --- |
| 0 | 0 | a |
| 1 | 0 | b |
| 0 | 1 | c |
| 1 | 1 | d |

**Legend:** Example of calculation of the interaction between a treatment within a specific interval and a resistant culture within a specific interval by calculating (*d*−*c*) − (*b*−*a*). 0 and 1 indicate the absence or presence of any treatment or any resistant culture within the specified intervals. The process is repeated for antibiotic treatments and susceptible positive urine cultures and between treatments at differing time intervals.

**Table S2: Summary of antibiotic resistance by organism**

| **Organism [N, (%)]** | **Cephalosporin** | **Fluoroquinolone** | **TMP/SMX** |
| --- | --- | --- | --- |
| *Escherichia coli* [213,833, (54.5)] | 31,761 (14.9) | 58,620 (27.4) | 52,475 (24.6) |
| Klebsiella spp. [83,016, (21.1)] | 13,500 (16.3) | 7,011 (8.5) | 10,977 (13.2) |
| Proteus spp. [37,466 (9.5)] | 5,878 (15.7) | 9,855 (26.3) | 8,991 (24.0) |
| Enterobacteraceae* [36,517, (9.3)] | 14,284 (39.1) | 3,709 (10.2) | 4,833 (13.2) |
| Citrobacter spp. [15,080, (3.8)] | 3,719 (24.7) | 819 (5.4) | 1,410 (9.4) |
| Serratia spp. [6,735, (1.7)] | 1,967 (29.2) | 352 (5.2) | 240 (3.6) |

**Legend:** *Enterobacteraceae can be further broken down into Enterobacter spp. (51.9%), Morganella spp. (16.5%), Providencia spp. (15.7%).

**Figure S1:** Histogram of predictions on the testing set conditioned on truth (0 = susceptible, 1 = resistant). The y-axis refers to the number of predictions within each x-axis bin.





**Figure S2: ROC (Left) and Precision-Recall (Right) Curves for each predictive model.**


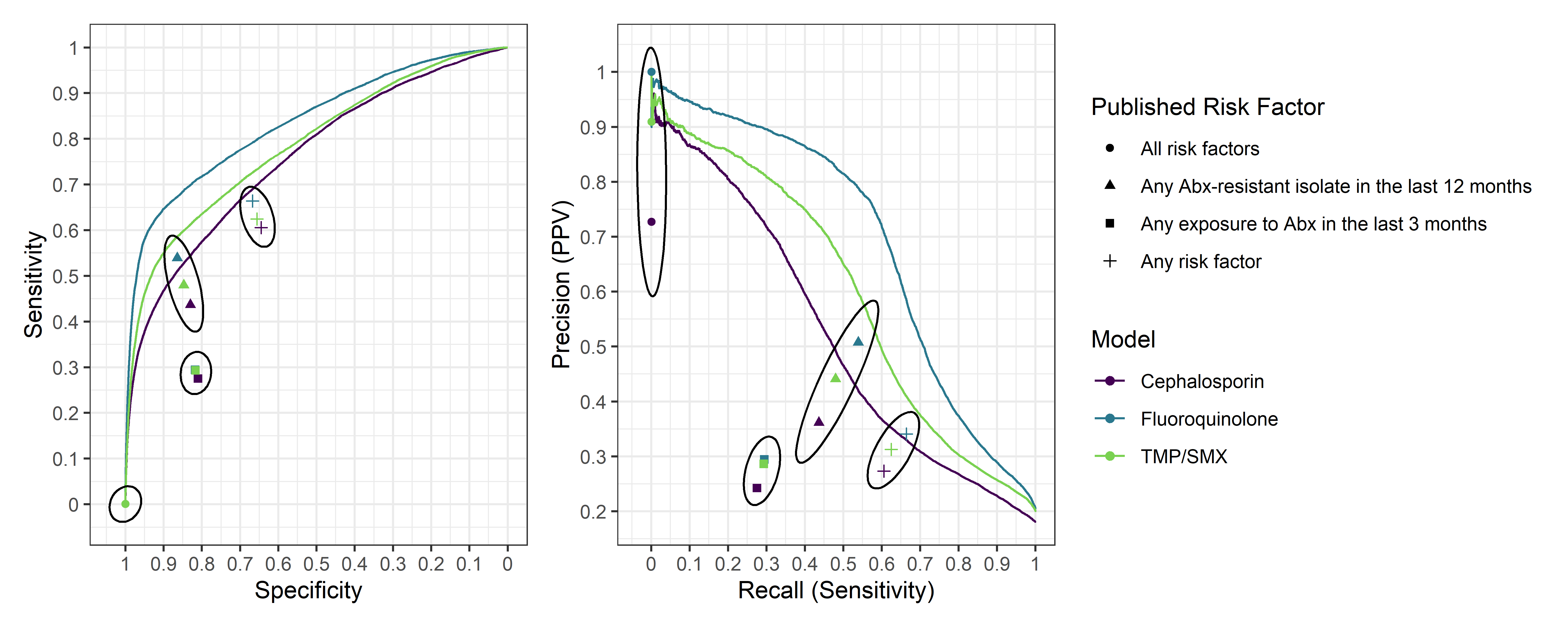


**Legend:** The plots show the trade-off between sensitivity and specificity (Left), precision and recall (Right). Individual points represent performance of referenced risk criteria for exposure or isolate specific to any or all drug types based on shape.

**Figure S3: Calibration curves for assessing moderate calibration and estimates of the weak calibration intercept and slope (top left).**


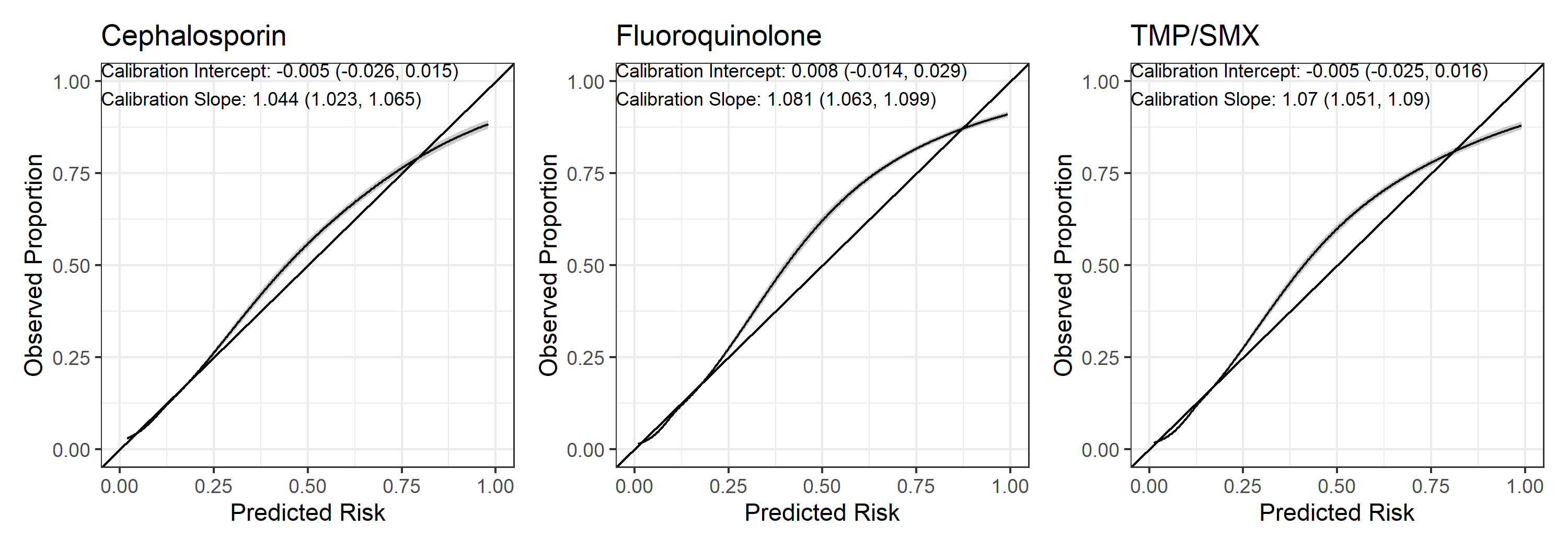


**Legend:** Each intercept 95% confidence interval contains 0 and the calibration slope point estimates and 95% CI’s exceed 1, suggesting a tendency to predict slightly lower than the observed event rate. This same tendency is shown when assessing moderate calibration by fitting a smooth curve to the predicted risk vs. observed proportion.

**Figure S4: Plot of individual SHAP values (y-axis) for prior antibiotic exposure and resistance by jittered value of that feature (x-axis) with average value as larger point.**


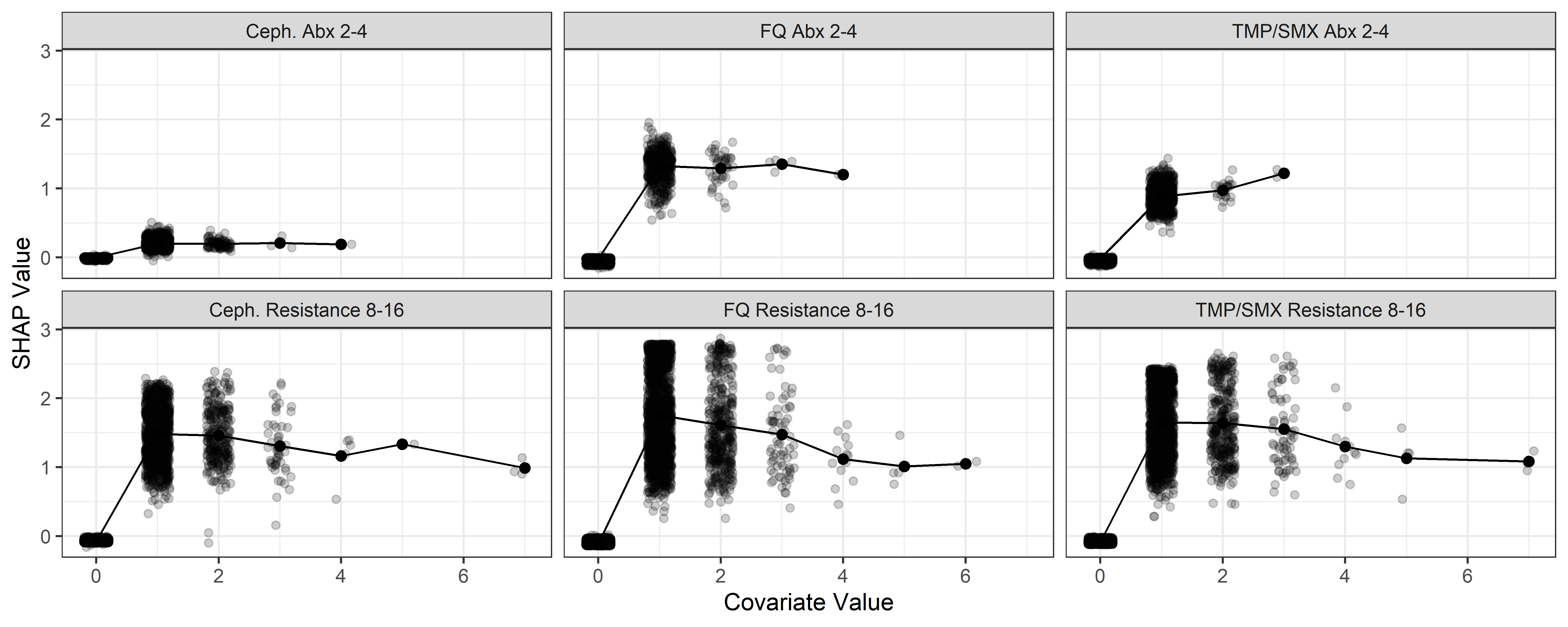


**Legend:** The top row of facets contains the SHAP values for the feature based on antibiotic treatment 2-4 weeks prior and the bottom row of facets contains the SHAP values for the feature based on having a resistance urine culture 8-16 weeks prior, each for subsequent resistant cultures.

**Figure S5: Heat plot presenting the assessment of interactions between a history of resistant or susceptible urine cultures and having had an antibiotic treatment using difference in average SHAP values.**


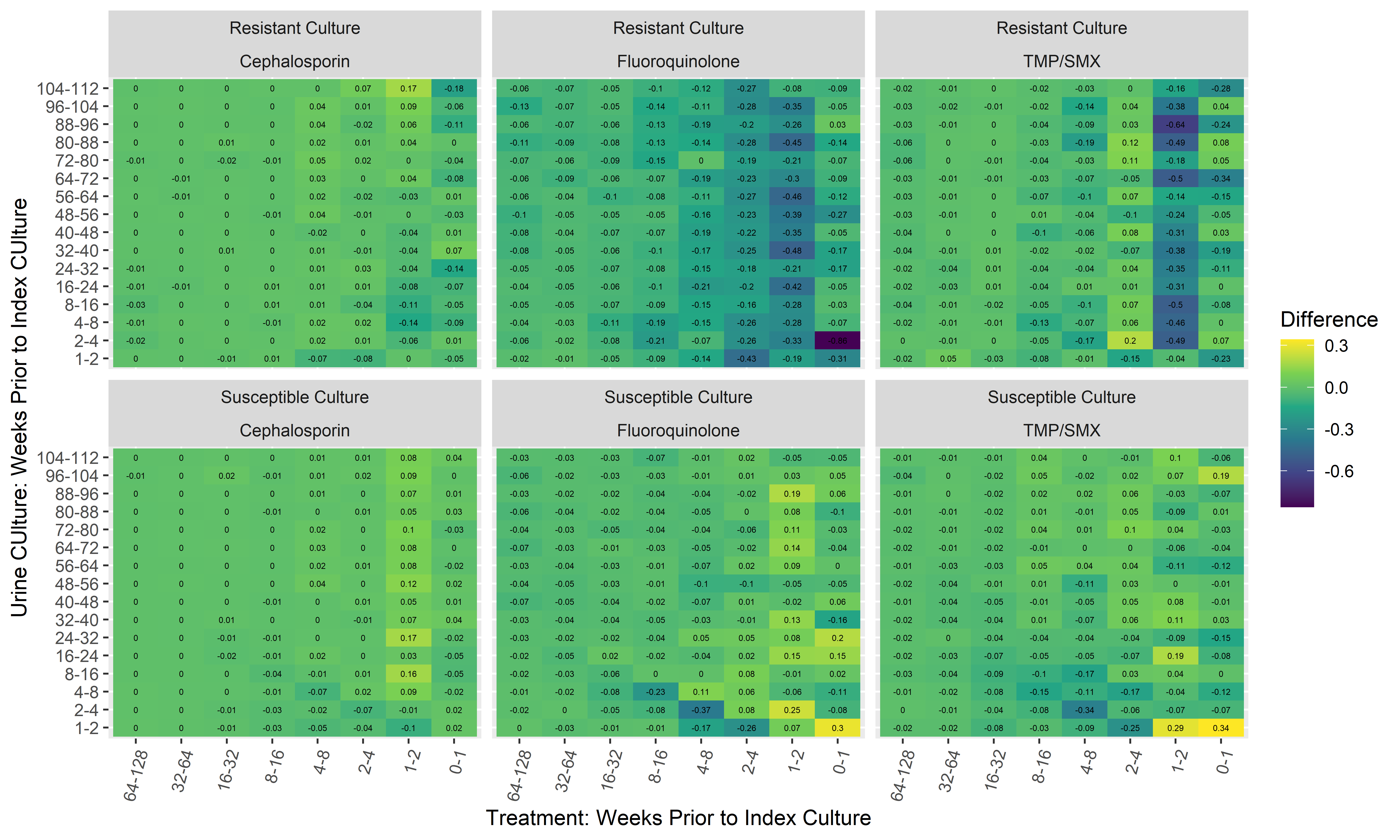


**Legend:** Interactions between difference in average SHAP value for prior antibiotic exposure versus not exposure over time (x-axis), according to whether or not there is a history of an antibiotic resistant culture (Top) or susceptible culture (Bottom) by weekly interval (y-axis). The color represents the magnitude of this difference, i.e. the interaction effect. Blue suggests that the effect of the treatment on the prediction is lessened among individuals who have a positive culture history. In some circumstances, for individuals who have resistant organisms, the effect of treatment on prediction is lessened. The opposite is true for those individuals who have a susceptible culture history. E.g., the -0.86 in the top middle panel at the 1 on the x-axis and 4 on the y-axis suggests that the difference in average SHAP value between those having received FQ treatment in the last week and not decreases by 0.86 in those who also had a urine culture resistant to FQ 2-4 weeks ago.
